# The Treatment of the Chronic Osteomyelitis of War (Based upon the Observation of 300 Cases)

**Published:** 1918-03

**Authors:** 


### ABSTRACT

***The Treatment of the Chronic Osteomyelitis of War (Based upon the Observation of 300 Cases).*** By M. H. Louis ROCHER, Surgeon of the Orleans District and Professor of the Faculty of Bordeaux. Trans. and Abs. [from the *Archives de Médecine et de Pharmacie*, June, 1917.

R. notes two types of chronic osteomyelitis : one, a subacute traumatic osteomyelitis (in the case of lesions one or two months old); and the other, fistulous osteomyelitis with a callus of long standing, often hypertrophic or otherwise abnormal (occurring in old wounds, some dating from the beginning of the war). He classifies methods of treatment for the prevention of such prolonged suppuration of the bone, as *radical* and *conservative*, the difference consisting in the extent of excision to remove bone splinters. The *radical* method, of which Leriche is an advocate, call for the excision of all splinters both attached and detached, and a wide opening of the medullary region for purposes of drainage. The author agrees that thorough drainage and cleansing of infection is necessàry, but raises against the radical method the danger of pseudarthrosis. He does not believe that the removal of the adherent splinters is necessary to avoid osteomyelitis or enlarged callus.

These difficulties may be prevented by careful cleansing of the region of the fracture, by the removal of devitalized tissues, detached splinters and all foreign matter, and by the use of antiseptic solutions to prevent ostitis.

The author would not, however, overlook the seriousness and great variety of bone infections. He urges the greatest thoroughness in localizing bone splinters, foreign matter, and fistulous trajects. Mere radioscopic examination is sufficient only in the actual process of removing an object. Careful radiographs should be made (face and profile) before the operation begins and in some cases with an instrument or bismuth inserted, so as to make sure of complete access, especially in long and circuitous trajects.

It is likewise essential that exploration and operation should be carried on with complete electric lighting, otherwise the presence of ostitis and infection centers may be overlooked.

*Treatment of subacute traumatic osteomyelitis.* — Often a fracture of one or two months' standing, which was treated at the front by a considerable excision of splinters, appears to be doing well except that a thin yellowish discharge continues from the wound made by the projectile or by some instrument, although there may be none of the usual signs of inflammation. This pus may indicate the presence of free splinters, foreign matter, or necrosing ostitis of the bone itself or of adherent splinters. There may also be subacute medullitis. A supplementary excision of splinters, following as nearly as possible the path of the original operation, should be attempted at once. The bony tissue should be curetted until healthy tissue is reached, even if this involves the removal of adherent splinters. Sometimes necrosed fragments are to be found deeply imbedded in the medullary canal. Bony fissures should be explored for possible infection, and, in short, every precaution taken to prevent further necrosis or ostitis. It should be noted, however, that radiography, as well as the results of actual operations, indicates that osteomyelitis is not limited in the medullary canal by bony barriers, and that it is therefore useless to empty this canal for the sake of reaching such barriers. This operation in itself may be attended with serious consequences, among them a temperature of 39°C. for about a week. The operator should be on his guard against all the possible complications in the bone tissue and its environment, such as, rarifying or condensing ostitis, fatty degeneration, decalcification of the bone, and congestive or purulent marrow. The most favorable time for such secondary operation is from six to eight weeks after the first, because the pathological conditions are then sufficiently definite to be clearly detected and remedied.

*The Treatment of chronic, fistulous osteomyelitis.* — This affection generally appears in cases six months or more old, many of which have already undergone various treatments, surgical or otherwise. Even after infection has apparently ceased, it may return as a result of injury or fatigue. This is likely to be one of the most serious afflictions after the war.

It is generally due to some of the following causes, hidden fragments, adherent splinters, that have become devitalized (these may be recognized by their edges and their smooth surfaces), late formations upon the bones resulting from the progressive necrozing ostitis (these may be recognized by their fringed edges and rough surfaces).

*Technique of operation for chronic osteomyelitis.* — Provisory hemostasis suffices if any is needed, but no bandaging at all offers the best opportunity for exploration, and is often practicable if there is a competent assistant to sponge the depths of the cavity.

Usually an *approach* to the surgical face of the bone is best. Care should be exercised to avoid great veins and arteries or important nerves. If these are exposed they may be seriously injured in the drainage or subsequent treatment of the wound. In the case of slight wounds it is enough to follow the fistulous duct : in more serious ones, the operation must be controlled by radiographs; but as the bone itself does not furnish sufficient indication of the conditions, it is best to insert an instrument or bismuth in the wound. In deep wounds the quickest and least destructive approach is desirable.

In case it is thought best to follow the path of the earlier operation, all of the wound tissue including that of the periosteum should be separated by the rugine from the seat of the osteomyelitis. Fungous growths indicate the entrance of the ducts leading to the infected centers. The work of the rugine should be followed by excision of fibrous tissues bordering upon the opening. Guided by the probe and radiographs the operator should gradually make a large opening in the bone, in the meantime removing all periostosis, and thus lay bare the seat of disturbance. In difficult cases it is well to operate upon a radioscopic table to make sure of complete cleansing. No possible opening into an infected pocket should be left unexplored. The operation should not be hurried. It is well to explore twice the surface of the cavity. Care should be exercised to distinguish between spongy tissues containing pus and infection and those simply congested or rarified by the infusion of marrow fat. This distinction is often difficult to make and requires a careful assistant to sponge the wound, and a mirror to assist in illumination. A mistake of this kind often leads to a much larger sacrifice of healthy substance than is necessary, and is often the cause of " formidable " excisions made by the radical school. The author believes that as much bone as is absolutely necessary should be removed, but no more.

The filling of the bone cavity may be accomplished by slow spontaneous growth, which requires in some cases several years; or it may be protected by periosteal cutaneous flaps or by periosteal muscular ones. These may be inserted in the cavity after detachment, and held in place by a tight bandage. The spontaneous growth method is the only one that may be employed for cavities in the epiphyses of large bones, of short bones (such as the calcaneum), of the iliac, or of the sacrum. The author declares that to this method of healing three fourths of his results are due in the case of diaphyseal and epiphyseal cavities.

The quicker method of periosteal skin flaps may be employed where the bone cavity is shallow throughout; and a muscular insertion may be made where the bone is completely surrounded by muscles (femur, humerus). Immediate suture is then possible if opportunity for drainage is allowed.

*The treatment of abnormal osteomyelitic calluses.* — In the case of an old fracture united in bad position, accompanied by chronic fistulous osteomyelitis, excision must be followed by the correction of the position. If as a result of the excision the diaphyseal extremities are almost freed, it is a comparatively simple matter to perform manual osteoclasis, osteotomy, or extension by pullies. A Lambotte plate or bone suture is sometimes necessary. It should be remembered, however, that sometimes, even after all signs of infection have disappeared, small fungous pockets may lie concealed and for a long time the bacteria remain dormant. Osteotomy of the callus will often revive them, and thus a bone suture or graft may be unsuccessful.

*Treatment of false joints with osteomyelitis.* — It is not intended to consider false joints caused by loss of bony tissue but simply those with flexible fibrous fistulous callus, which is both long and soft, and which calls for special treatment. The author has in mind especially those cases in which an abnormal periosteal formation renders it impossible for the diaphysary extremities to unite in a solid callus.

The chief aim of the operation is to remove all infection and causes of infection. The operator should be on his guard against fibrous tissue which may represent a bit of muscle devitalized by a bone fragment, and also against adherent splinters that may be so placed as to prevent bone growth. The cleansing once completed, the bones should be brought together and immobilized or sutured. If thought best immobilization may be accomplished by winding with metal wire, or colored catgut, or a Lambotte plate. These last should be removed at the end of the 25th day if the union is sufficiently firm, and a plaster appliance substituted. It should be remembered that wire and screws may be broken by strain or by oxidation.

*Treatment of fistulous bone growths of the periosteum.* — These result commonly from projectiles that graze the bone, irritating or actually penetrating the periosteum. As a result of the disturbance the periosteum constructs new bone tissue in the region of the lesion. The growths are distinguished from those attending osteomyelitis by their shape and size. They assume various forms, as of a cauliflower, a large spur, an oval mass, a formless block, a bony accumulation, or a sheet of bone perforated in such a manner as to invite fungous nests. Again it may appear as a bony trail following the tract of the projectile. It may be made up of compact or spongy bone tissue or it may consist of a mixture of both. An instrument introduced into the fistulous duct strikes upon the bare bone.

Surgical treatment is necessary in all such cases. The bone tumor should be completely removed by large gouge forceps or scissors. The operation should be followed by the most minute cleansing of all fungous regions.

*Treatment of chronic osteomyelitis complicated by nerve lesions.* — Surgeons and neurologists agree that in case of serious nerve lesions resulting from osteomyelitis, it is best to operate if there is no improvement at the end of three months. If the injury consists merely of compression, tension, or irritation caused by exostosis or splinters, operation surely gives relief. If there is partial or complete section of the nerve, the results are very doubtful. The obstacles to successful suture are almost unsurmountable. The presence of infection often defeats suture in spite of any possible muscular protection. Chronically inflamed tissues and considerable loss of substance, make it often impossible to liberate the nerve ends or to bring them together. This is even more difficult when the search requires cutting of the bone.

*Treatment of osteomyelitic cavities after sterilization of scar formation.* — Some of the cavities due to loss of bone substance, especially in the diaphysis of the femur or the upper extremity of the tibia, fail to develop complete epidermization, and even when a satisfactory scar is formed, an uncomely gap results.

When a sufficient scar is not formed, it is best to graft with skin, or else to bring down over the gap the cutaneous flaps. These grafts should be fixed in place by means of a tight bandage over the bone cavity. The author has tried muscle grafting under difficult circumstances without much success, but believes that it would give esthetic results if performed after the wounds are healed, and all danger of infection avoided.

*Post-operative complications.*

1. *Return of infection.* — This may take the form of an acute attack of osteomyelitis, pandiaphysitis (due to faulty operation that has inoculated the medullary canal), or subacute or suppurative arthritis (when the gap is near a joint). In some cases, in spite of all that may be done, amputation is the only resort.

2. *Bending or fracture of the weakened bone.* — The first of the consequences is due to a failure of the bone to withstand the powerful action of the great muscles. This may be overcome by several days of extension in a plaster appliance.

The seriousness of a resulting re-fracture with a possible return of acute osteomyelitis, should lead the operator to use a grooved or plaster splint during the healing of any bone that has been considerably weakened by surgical cleansing.

3. *Persistent suppuration.* — A secondary destructive ostitis often results from an incomplete operation, irregular and defective bandaging, or faulty drainage.

4. *A painful callus*, sometimes of considerable size, may persist in spite of a normal healing of the bone cavity. The cause of this affection is far from clear. Nerve fibres may be compressed or strangled by intra-osseous condensing ostitis. In such cases no further surgical treatment should be undertaken until thermal and physiotherapeutic means have been tried for a long time.

5. *Infected Callus* is the most frequent cause of post-operative pain. A latent infection, reviving sometimes long after a patient is considered cured, may give rise to ostitis in the region of the fracture. The scar swells and pus appears through some reopened duct.

*The technique of bandaging for chonic osteomyelitis.* — Immediately after the operation a *tight* bandage should be applied. The wound caused by the operation should be filled with tightly packed gauze to prevent the blood from soiling the bandage and the bedding. In some cases of profuse bleeding it is well to remove the bandage and inspect for breaks in important arteries, some of which may result from drainage. Ordinarily, however, it is unnecessary to reopen. By tightening the superficial bandage the flow of blood may be sufficiently stemmed.

According to the indications of the bone cavity, the gauze pack may be saturated (but well wrung out) with hydrogen peroxide, Dakin solution, ether, or iodoforme. *Renewal of the dressing*, in cases where a slight degree of infection may be present, should take place at the end of 48 hours. From this time on the dressings should be changed daily. The bandages may be saturated at the time of dressing with Dakin or hydrogen peroxide, or kept moist by means of the intermittent irrigation method (Dakin, hypertonic serum, permanganate of potash). These dressings should be continued until all suppuration has disappeared.

In case of lesions with little infection, either ether or iodoform, changed at the end of four or five days, is preferable. After the first change, the frequency must be regulated by the reaction, the interval varying from every three to every six days. If blue pus appears, cyanide of mercury (1 : 1000) makes an effective dressing.

In the course of the second week following the operation, granulations develop both at the bottom and on the sides of the bony cavity. Every opportunity should be given them to develop evenly; and to this end, the healing of the skin should be carefully avoided, because it prevents proper drainage and encourages reinfection. If any infection appears, the lips of the wound should be kept wide open, and packs applied, gradually yielding their place to the granulations. Only in this way may regular and sure healing take place.

As soon as all suppuration has disappeared, daily dressing may be abandoned, and infrequent dressings made with iodoformed ether, Calot's solution, Mencière's solution, Reclus pomade, or simple sterilized gauze.

In any case, gauze should not be continued indefinitely in the bony cavity, for it retards growth. When the fistulous tract does not disappear spontaneously, small infiltrations by means of Pravaz's syringe using chloride of zinc, silver nitrate, Beck's bismuth paste, or iodargol, have brought about the desired results.

After the gauze packs are removed capillary drainage by Florence hairs has been used for a while with good results. Exposure to the sun may hasten repair.

In the case of the smaller bones, healing under good conditions requires about two months; but for large bones, from three to four months. It should not be forgotten that success depends quite as much upon the skill of dressing and bandaging as upon the operation itself. This work should not be entrusted to any but physicians skilled in bone surgery, or to nurses who have been carefully trained by them.

*Hydro-mineral treatment.* — Those who recommend thermal treatment during the period of convalescence at such stations as Barèges and Salies de Béarn, claim that it is effective in hastening elimination of devitalized bone and projectiles by expulsion, also that it aids healing, and prevents congestion (cure of edema, periarticular infiltrations, intra-articular discharges, and defects of circulation). The author believes that elimination by this treatment is to be thought of only when all surgical treatment has failed, but he believes that in the matter of healing and congestion, thermal treatments may be most beneficial, especially if undertaken shortly after operation.

*Le Gérant :* O. Porée. Paris — Imp. Lahure, 9, rue de Fleurus.

